# Structural Characteristics of the Si Whiskers Grown by Ni-Metal-Induced-Lateral-Crystallization

**DOI:** 10.3390/nano11081878

**Published:** 2021-07-22

**Authors:** Béla Pécz, Nikolaos Vouroutzis, György Zoltán Radnóczi, Nikolaos Frangis, John Stoemenos

**Affiliations:** 1 Centre for Energy Research, Institute for Technical Physics and Materials Science, EK MFA, Konkoly-Thege Miklós út 29-33, 1121 Budapest, Hungary; gy.radn@mfa.kfki.hu; 2 Department of Physics, Aristotle University of Thessaloniki, 54124 Thessaloniki, Greece; nikosv@auth.gr (N.V.); frangis@auth.gr (N.F.); stoimeno@auth.gr (J.S.)

**Keywords:** crystallization of silicon, transmission electron microscopy, Moiré fringes

## Abstract

Si whiskers grown by Ni-Metal-Induced-Lateral-Crystallization (Ni-MILC) were grown at 413 °C, intentionally below the threshold for Solid State Crystallization, which is 420 °C. These whiskers have significant common characteristics with whiskers grown by the Vapor Liquid Solid (VLS) method. The crystalline quality of the whiskers in both methods is the same. However, in VLS, a crystalline substrate is required, in contrast to the amorphous one in Ni-MILC for the growth of single crystalline whiskers. Moreover, whiskers grown by VLS have a polygonal cross-section with their diameter determined by the diameter of the hemispherical metallic catalysts. On the other hand, in the Ni-MILC, the cross-section of the whiskers depends on the size of the NiSi_2_ grain from which they are emanated. This was confirmed by observing the crossing whiskers and the rotational Moiré patterns in the crossing area. The structure of disturbed short and thin nonlinear branches on the side-walls of the whiskers was studied. In the whiskers grown by the VLS method, significant contamination occurs by the metallic catalyst degrading the electrical characteristics of the whisker. Such Si whiskers are not compatible with the current CMOS process. Whiskers grown by Ni-MILC at 413 °C are also contaminated by Ni. However, the excess Ni is in the form of tetrahedral NiSi_2_ inclusions which are coherent with the Si matrix due to the very low misfit of 0.4% between them. These whiskers are compatible with current CMOS process and Thin Film Transistors (TFTs).

## 1. Introduction

Polycrystalline silicon (poly-Si) films are used in a wide range of applications, such as large-area electronics, including Thin-Film Transistors (TFTs), solar cells and sensors. Solid-Phase-Crystallization (SPC) is one of the simplest methods to crystallize amorphous silicon (a-Si) films, though it requires temperatures above 600 °C [1,2]. In most applications, the substrate is low-cost soft glass which requires lower process temperatures. The crystallization temperature of a-Si can be lowered by the Ni-Metal-Induced-Lateral-Crystallization (Ni-MILC). For the MILC, the preferred metal up to this date has been nickel (Ni) due to its low residual metal contamination in the poly-Si region [3]. In this case, the a-Si crystallized temperatures can be as low as 413 °C under the presence of nickel-disilicide, NiSi_2_. The crystallization temperature of a-Si lowers under the presence of NiSi_2_ because Ni atoms from the NiSi_2_/a-Si interlayer diffuse into a-Si, reducing the strength of the covalent bonds at the interface caused by their interaction with the free electrons from the metallic phase [4].

At the relatively low temperature of 413 °C, whiskers are only grown by Ni-MILC, which makes them worth comparing to those ones grown by the standard VLS method, [5,6]. In the VLS process, the seed for the growth of the Si whisker is a droplet of liquid metal-Si alloy, in most cases this is Au-Si alloy on a (111) Si substrate. The liquid droplet is a preferred site for the Si deposition from the vapor phase; hence, the droplet becomes supersaturated with Si. Subsequently, the excess Si precipitates on the leading face of the Si whisker at the backside of the droplet, resulting in the growth of the Si whisker [5]. In both methods, long single crystalline whiskers are formed. In the VLS case, the isolated whiskers are perpendicular to the substrate, in the MILC case, they are parallel to the substrate and surrounded by a-Si.

In the case of Ni-MILC, the seeds are NiSi_2_ crystallites which are formed after the reaction of Ni with the a-Si film at temperatures as low as 250 °C [7]. The NiSi_2_ is cubic, having the CaF_2_ structure and lattice mismatch with crystalline Si of only 0.4%. During annealing, nickel atoms from the NiSi_2_ seeds diffuse into a-Si, forming new layers of NiSi_2_ and leaving behind vacancies, accumulated at the backside of the NiSi_2_ grain. Subsequently, a diamond type rearrangement of the Si bonds occurs, resulting in an epitaxial Si layer at the backside of the NiSi_2_ module. This continuous process forms Si whiskers, as described in detail in Refs. [8,9]. In this study, we will show that whiskers grown in a-Si by Ni-MILC at 413 °C are similar to those grown by the VLS process. It is worth noticing that the residual Ni contamination of the Si whiskers grown by Ni-MILC is significantly lower in respect to those grown by VLS, where the metal impurity is gold, which is detrimental for electrical properties [10].

We want to note that at high temperature (typically 600 °C), MILC process, also Solid State Crystallization (SPC), happens in a significant way. Namely, the Si whiskers act as seeds facilitating the SPC crystallization around the whiskers (this will be shown later). As this second growth (SPC) process around the whiskers occurs spontaneously, large, highly defected crystallites are formed. The process is described widely in the literature, while the extremely low-temperature case where SPC is completely avoided is worth studying as well.

In order to study the influence of the SPC in the Ni-MILC process, annealing was performed at 600, 555, 490, 454, 420 and 413 °C. In each case, the contribution of the SPC was estimated by Transmission Electron Microscopy (TEM) observation of the mosaic structure development around the whiskers. It was shown that even at 454 °C, the influence of the SPC was noticeable; below 420 °C, no influence of the SPC was observed. These results are included in our previous papers, Refs. [7,11,12]. This should be considered as the threshold of the SPC in Ni-MILC. In the present work, we have studied the structural characteristics of whiskers grown below this threshold, namely at 413 °C, which corresponds to the lowest temperature annealing we have performed. In this manner, we could study the formation of Si nanowires, which is the characteristic of Ni-MILC process, and compare the results to vertical Si nanowires grown on single crystalline silicon by the Au-VLS method.

## 2. Materials and Methods

**Figure 1:**
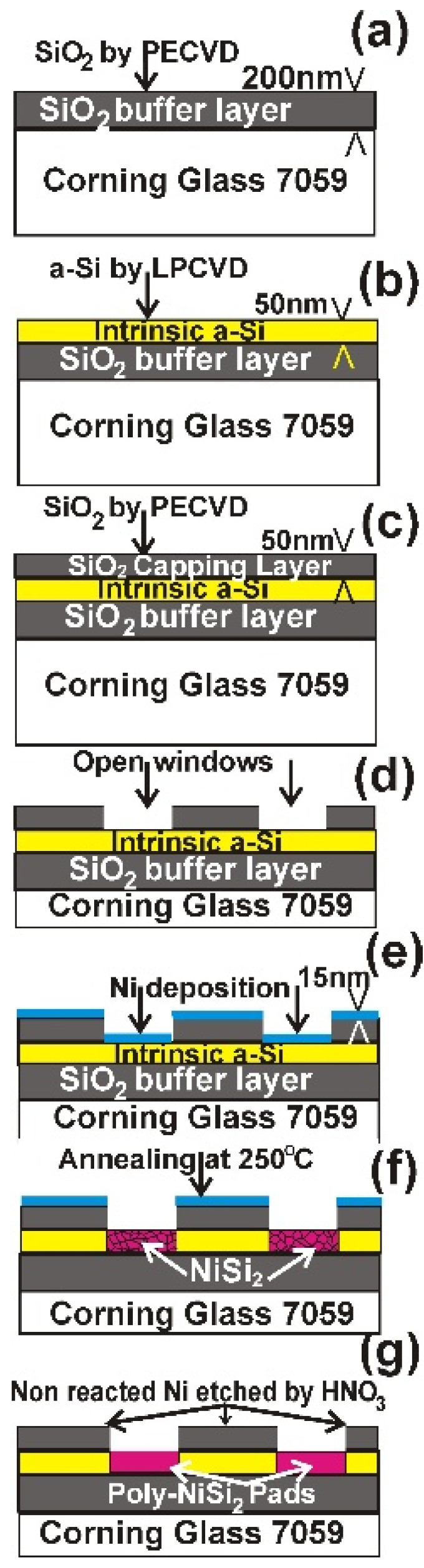
Schematic representation of the specimen preparation for the Ni-MILC experiment at 413 °C. (**a**) Deposition of 200 nm thick SiO_2_ buffer layer on glass substrate by PECVD. (**b**) Deposition of 50 nm a-Si by LPCVD. (**c**) Deposition of a 50 nm thick SiO_2_ capping layer by PECVD. (**d**) Open windows in the SiO_2_ capping layer for the pad formation. (**e**) Deposition of 15 nm thick nickel. (**f**) Annealing at 250 °C for 10 min in order to form polycrystalline NiSi_2_ in the area of the windows. (**g**) The nonreacted Ni is etched by HNO_3_.

The specimen preparation for the Ni-MILC experiment at 413 °C was already presented in detail in Refs. [11,12]. A brief presentation of the procedure is shown schematically in Figure 1. The specimens were annealed at 250 °C for 10 min in nitrogen atmosphere for the formation of NiSi_2_ pads, as shown in Figure 1f. The nonreacted Ni was etched by HNO_3_, (Sigma Aldrich, Athens, Greece) Figure 1g. The thickness of the deposited Ni film was chosen to give stoichiometric NiSi_2_ to all the depth of the a-Si film. In this way, a pattern of NiSi_2_ pads was formed. Annealing was performed for the realization of the Ni-MILC process. For this purpose, samples with dimensions of 15 mm × 6 mm were placed in quartz ampoules which were sealed in vacuum {8 × 10^−2^ (Pa)} and annealed at 413 °C for 11 Ds (days), and for 32 Ds. Annealing was performed in an oven equipped with a temperature controller; moreover, a thermocouple was also used to check the actual temperature of the specimens. The accuracy of the temperature measurement is ±1 °C. Conventional MILC specimens were annealed at 520 °C for 1 h in nitrogen atmosphere for reference purposes. Specimens for Plane View TEM (PVTEM) observations were prepared by etching the capping protection SiO_2_ layer, the SiO_2_ buffer layer and the glass substrate using HF (Sigma Aldrich, Athens, Greece) and subsequently lifting off the Si film on gold micro-grids. For the structural characterization, a 2010 JEM microscope (JEOL Corp. Tokyo, Japan) as well as a Thermo Fisher THEMIS 200 image corrected microscope (Eindhoven, The Netherlands) was used.

## 3. Results and Discussion

A Si whisker grown by Ni-MILC at 413 °C is not affected by Solid Phase Crystallization (SPC) [7,11]. In contrast, the same process in the range of temperatures 500–600 °C is strongly affected by SPC, creating a high density of defects. Therefore, the absence of SPC at Ni-MILC at 413 °C results in better quality Si whiskers comparable to those grown by the VLS method.

At first glance, the contribution of SPC in Ni-MILC should be insignificant because the incubation period for random nucleation at 600 °C is more than 10 h [2]. However, the incubation period for a-Si crystallization in Ni-MILC is zero, as was shown by an in situ TEM experiment, Radnoczi et al. [7]. Therefore, SPC is significant in Ni-MILC because the pre-existing whiskers act as seeds for crystallization unless the annealing temperature is sufficiently low to prevent the crystallization.

**Figure 2:**
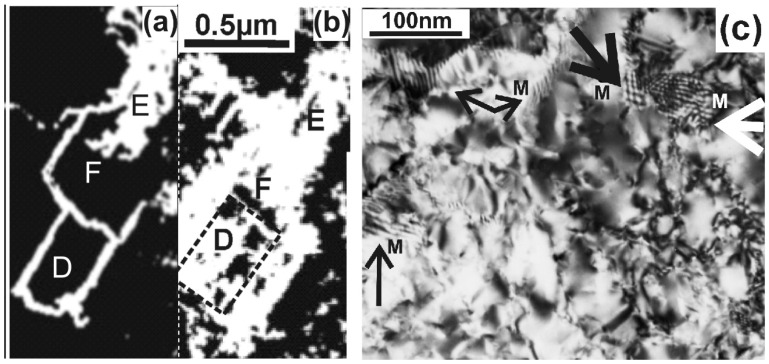
TEM micrographs from in situ Ni-MILC at 520 °C where SPC is significant. (**a**) In the areas D and F, Si whiskers are evident, frequently changing direction, forming a loop in area D. (**b**) The same area 2 min later, the areas D and F are covered by slightly misoriented grains due to SPC. (**c**) Slightly misoriented overlapping grains due to the SPC process give Moiré patterns of rotational type shown by arrows.

The contribution of the SPC at 520 °C is shown in the TEM micrographs in Figure 2a,b. Figure 2a shows the formation of Si whiskers in the areas denoted by the letters D and F after annealing for 2 min. The same areas are shown in Figure 2b 6 min later. Now, the whiskers are surrounded by misoriented Si grains due to SPC using the whiskers as seeds. The significance of SPC in Ni-MILC above 500 °C is shown in the high magnification micrograph in Figure 2c where the whiskers are surrounded by slightly misoriented grains, resulting in a mosaic structure. Overlapping grains give a Moiré pattern of rotational type [13], which is denoted by the letter M in Figure 2c. More details on the SPC involvement in Ni-MILC above 500 °C are shown by M. Miyasaka et al. [14], also by Radnoczi, G.Z. et al. [7].

### 3.1. Tweed-Like Structure in Ni-MILC at 413 °C

**Figure 3:**
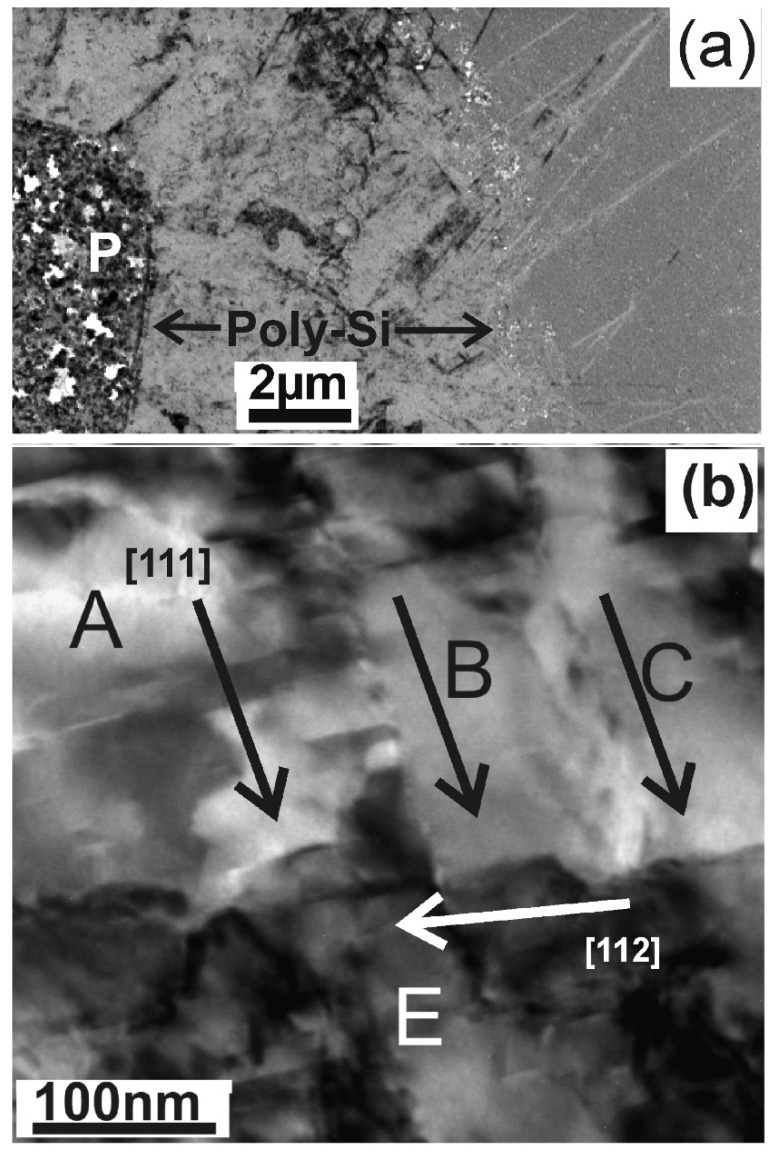
TEM micrographs from Ni-MILC at 413 °C where SPC is completely suppressed. (**a**) Overall view of the structure around the pad denoted by the letter P. A continuous poly-Si film consisting of a mixture of fast and slow whiskers resulting in a tweed-like structure is extended up to 8 μ. Then, long fast [111] type whiskers emanate due to growth–death competition mechanism. (**b**) The tweed-like structure shown at higher magnification.

The SPC process can be completely inhibited by lowering the annealing temperature to 413 °C, resulting in pure Ni-MILC. The films consist of a mixture of whiskers growing fast along the [111] crystallographic direction and whiskers grown slowly, having random crystallographic orientations, other than the [111] [5]. The overall view of such a film is shown at the low magnification micrographs in Figure 3a. The NiSi_2_ pad, denoted by the letter P, is surrounded by a net of fast [111] type and slow type whiskers, resulting in a tweed-like structure; this is shown in the high magnification macrograph in Figure 3b, where three parallel fast [111] whiskers, A, B, C, are intersected by the slow [112] whisker E. At the edges of the tweed-like film, bands of long parallel [111] type whiskers are observed as shown in Figure 3a. This is the result of the natural crystal filtering due to growth-velocity competition which is observed when grains meet other grains that have already been crystallized [13,14]. The observed long whiskers must be exactly parallel to the substrate; otherwise, they touch the surface or the bottom of the film and stop. Whiskers as long as 9 μm were observed, as shown in Figure 3a. The crystal filtering effect was also observed in the Ni-MILC at 550 °C, where SPC is also involved, by artificial preferred filtration through a narrow neck in the a-Si which was formed artificially by lithography, creating a SiO_2_ barrier in the a-Si, before the onset of Ni-MILC [15,16].

### 3.2. Crossover of the Whiskers in Ni-MILC at 413 °C

**Figure 4:**
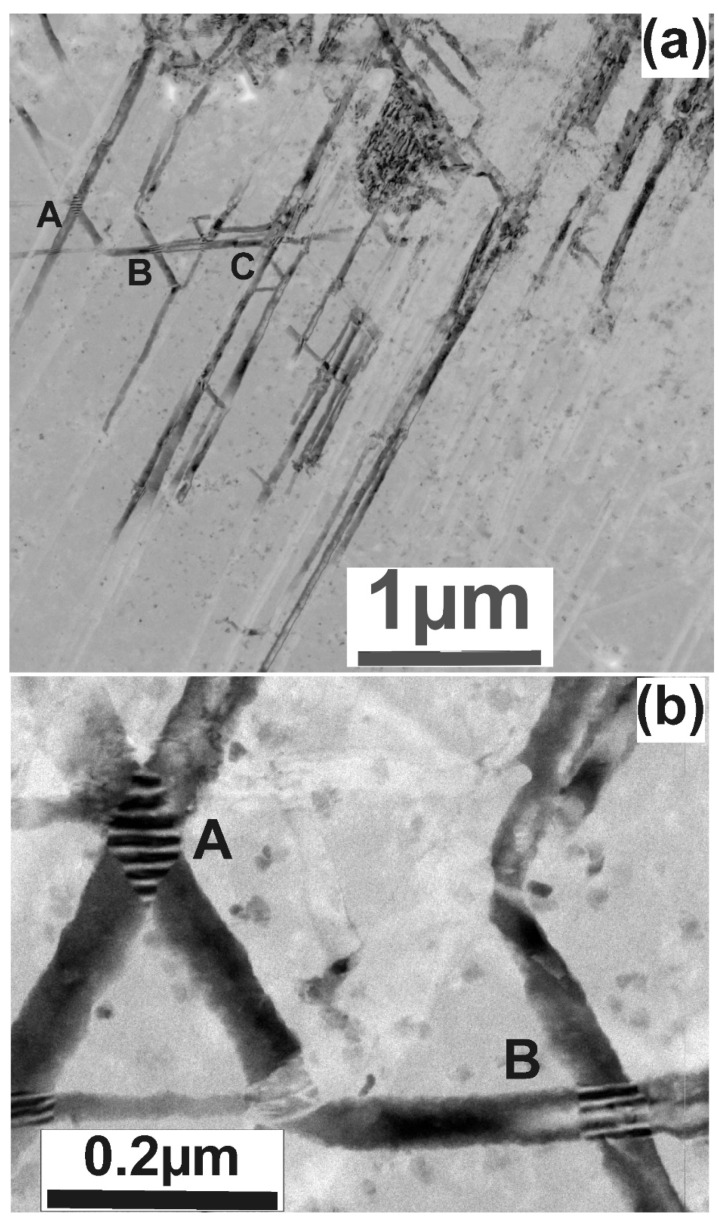
TEM micrographs. (**a**) Overall view of crossing whiskers; in some cases, the same whisker crosses several whiskers having different orientations denoted by the letters A, B and C. (**b**) The crossing areas A and B at higher magnification reveal the formation of Moiré fringes.

In some cases, the whiskers can cross each other, as shown in Figure 4a, denoted by the letters A, B and C. Very often, Moiré patterns are formed in the crossing area, revealing overlapping of the whiskers in this area. This is evident in the areas A and B, as shown in the higher magnification micrograph of Figure 4b.

**Figure 5:**
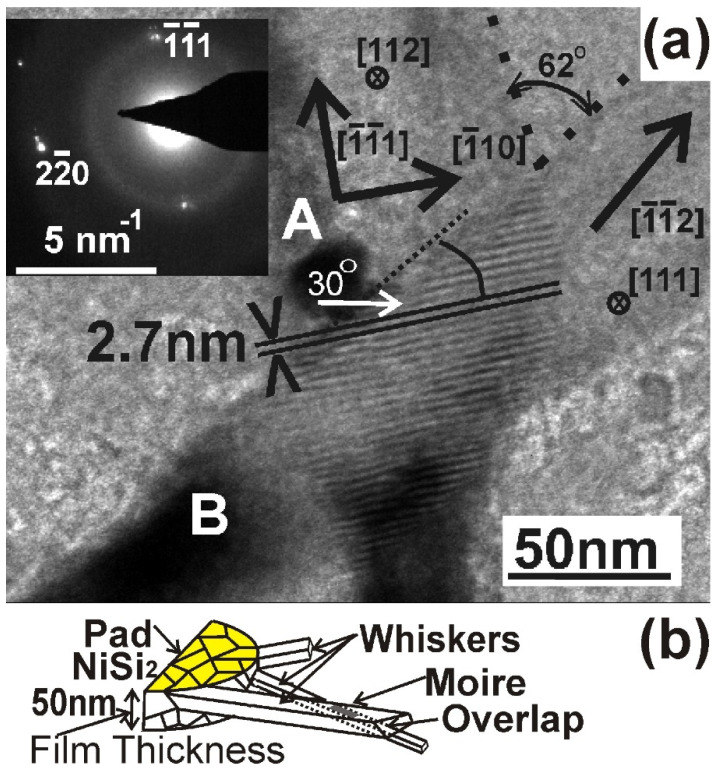
(**a**) High magnification TEM micrograph from the crossing area of two whiskers A and B in the inset is the corresponding diffraction pattern. The two whiskers have common reflection of 220, which is split; the Moiré fringes are perpendicular to this reflection, revealing that they are of the rotational type. (**b**) Schematic representation in 3D of two overlapping whiskers emanating from the NiSi_2_ pad. The total thickness of the overlapping whiskers A and B must not exceed the thickness of the film.

The formation of Moiré patterns in two crossing whiskers was systematically studied as shown in Figure 5a; the crossing whiskers are denoted by the letters A and B. The diffraction pattern in the inset of Figure 5a was taken from the crossing area and corresponds to the (112) zone, revealing that the whisker A grows along the [111] direction; in other words, it is a fast type whisker. The reflection 220 is common for both whiskers and is split, making a small angle of about 3.5°. These double spots create the Moiré patterns in the crossing area. The Moiré patterns are extended perpendicular to the **g**_220_ reflection, namely along the [111] direction. Therefore, the Moiré patterns are of the rotation type [13], having a periodicity of 2.7 nm. The direction of growth of the whisker B is the [112]. This was deduced considering the angle which it forms with whisker A and with the common direction [110]. These are 62° and 30°, respectively; only the [112] direction forms such angles with the [111] and [110] directions. Therefore, the whisker B is a slow type whisker.

A more accurate estimation of the misorientation of the two whiskers can be deduced by applying the equation

θ = d/D(1)

where D is the periodicity of the Moiré patterns, d the spacing of the lattice planes of the operating diffraction, in our case d_220_ = 0.192 nm and θ the angle of the misorientation in rad. For D = 2.7 nm, we have θ = 0.071 rad = 4.07°; this is the exact angle between the split 220 spots. The overlap of the two whiskers A and B is shown schematically in 3D in Figure 5b. The total thickness of the overlapping whiskers A and B must not exceed the thickness of the film, which is 50 nm.

**Figure 6:**
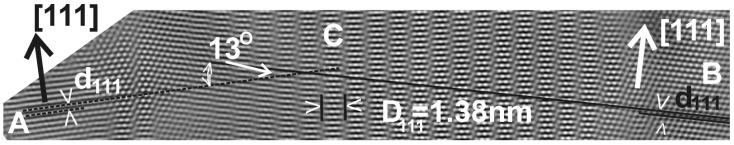
High-resolution TEM micrograph from two partially overlapping [111] type whiskers A and B; the overlapping area is denoted by the letter C. The observed rotational Moiré pattern has a periodicity of D_111_ = 1.38 nm.

According to Equation (1), when the overlapping whiskers form a relatively large misorientation angle θ, the periodicity of the rotational Moiré patterns is small, requiring high-resolution TEM to be revealed; this is shown in the high-resolution TEM micrograph in Figure 6. In this case, two fast [111] type whiskers A and B partially overlap, creating rotational Moiré patterns with periodicity D_111_ = 1.38 nm, as shown in Figure 6. The misorientation angle θ was calculated from Equation (1) with common reflection 111, (d_111_ = 0.3138 nm), resulting in θ = 13.035°. Since the whiskers A and B have the (110) zone axis, they also have the reflections 220 and 002 in common; therefore, rotation type Moiré should also be observed from these reflections. It is worth noticing that the 200 reflection is forbidden in the diamond structure; however, it appears, especially in the section (110) due to double reflection of the 111 and 111 spots; that is why it is denoted with a star (*). However, the Moiré patterns are intense only for reflections which are close to the “two beam” case, fading fast outside of it. This is the case in Figure 6, where only one set of Moiré patterns is observed.

**Figure 7:**
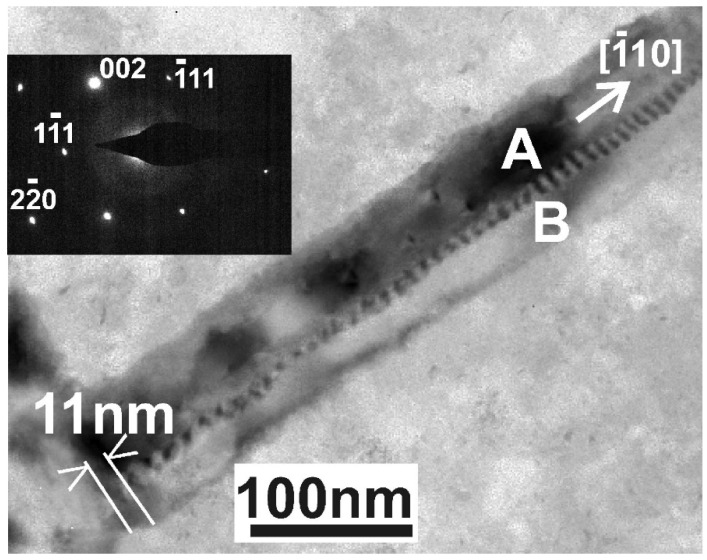
TEM micrograph of two partially overlapping parallel whiskers A and B. The related diffraction pattern in the inset reveals that the parallel whiskers A and B are in the (110) section, grown along the [110] direction, and therefore are of the slow type.

Another example of two partially overlapping parallel whiskers, A and B, is shown in Figure 7. The related diffraction pattern in the inset of Figure 7 confirms that the parallel whiskers A and B are of the slow type, grown along the [110] direction with their (110) planes perpendicular to the electron beam. The periodicity of the Moiré pattern is 11 nm, also running parallel to this direction, revealing that they are created from the strong 002 reflection which is perpendicular to the [110] direction. According to Equation (1), the angle of rotation θ for the reflection 002 is only 1.4°, too small to be distinguished in the diffraction pattern.

The cross-section of the whiskers grown by VLS are symmetric polygonal, growing from a single crystalline Si substrate [5]. In contrast, in the Ni-MILC, the Si whiskers grow from NiSi_2_ grains which have different size, shape and orientation. This explains why two whiskers having width of about 50 nm, as shown in Figure 5a, can overlap in a 50 nm thick film. Obviously, the cross-section of the whiskers in Ni-MILC is not symmetric polygonal, as shown schematically in the 3D Figure 5b. This is a significant dissimilarity in the two processes.

The Moiré patterns are very sensitive to any lattice misorientation. The exclusion of the SPC process results in homogeneous Moiré patterns in the overlapping whiskers. If the overlapping whiskers are also affected by the SPC process causing random small misorientations (Ni-MILC temperature above 500 °C), then the overlapping area would be divided into smaller areas, exhibiting rotational Moiré patterns of different periodicity and orientation due to the misoriented grains as shown in Figure 2c.

### 3.3. Width of the Whiskers

Whiskers grown by VLS have polygonal cross-section with a diameter determined by the diameter D of the hemispherical metallic catalysts according to the equation:

D = 4V_L_σ_LV_/RT ln(s) (2)

where V_L_ is the molar volume of the metallic droplet, σ_LV_ the liquid-vapor surface energy and s the degree of supersaturation of the vapor [10]. Nevertheless, Oswald ripening mechanism leads to the formation of larger droplets of metal catalyst. On the other hand, in the Ni-MILC case the cross-section of the whiskers depends on the size of the NiSi_2_ grain from which they are emanated. Thus, the mean size of the grains in the NiSi_2_ pads grown at 250 °C is 60 nm, but most of them are below 50 nm in the perpendicular direction as schematically shown in Figure 5b. The cross-section of the Si whiskers is fitted to the facet of NiSi_2_ grain from which they are emanated. Therefore, the whiskers are not symmetrical, as already schematically described in Figure 5b.

### 3.4. Saw-Tooth Faceting of the Side-Walls of the Whiskers

**Figure 8:**
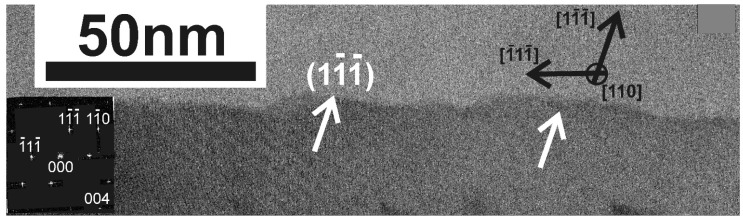
Cross-section TEM micrograph from fast [111] whiskers viewed in (110) section. A saw-tooth faceting of the side-walls is evident, with the longest segments of the tooth being the (111) planes.

In the fast <111> whiskers grown by Ni-MILC, saw-tooth faceting of the side-walls is frequently observed, with the longest segments of the tooth to be the (111) planes, as shown in Figure 8. Similar structures were observed in whiskers grown by VLS [5]. Saw-tooth faceting occurs when the side-walls are not stable; namely, they do not belong to the equilibrium crystal shape. In this case, the surface breaks into stable saw-tooth facets [17].

### 3.5. Straight Whiskers in Ni-MILC at 413 °C

**Figure 9:**
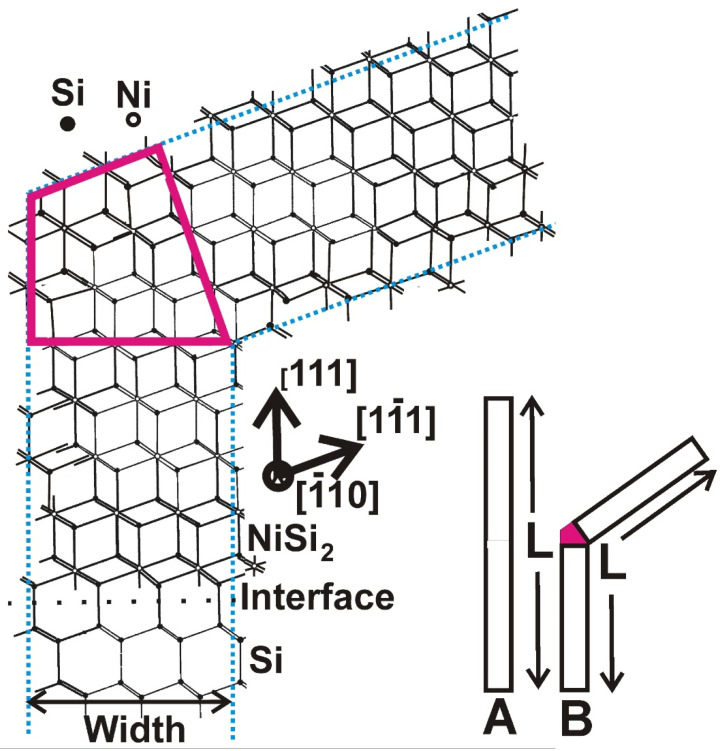
Schematic representation of a fast [111] whisker denoted by the letter A which grows linearly having a length L. Compare this whisker with the whisker B having the same total length L. During the growth, the whisker B switches to the equivalent [111] direction. Although the two whiskers have the same length, an extra part denoted by red is required for the switching, including the formation of extra dangling bonds. This is also shown schematically in atomic scale in the section (110).

In Ni-MILC above 520 °C, the fast [111] type whiskers prevail, changing their course frequently to other equivalent <111> directions, facilitating in this way the crystallization [13], as shown in the TEM micrograph in Figure 2a,b. In contrast, the whiskers in Ni-MILC at 413 °C are, in general, straight, rarely changing their course as shown in Figure 3a and Figure 4a. It is speculated that the reason for this difference is the extra dangling bonds which are required when a [111] whisker changes its course to another equivalent direction, say the [111] as schematically shown in Figure 9. The whisker A is a fast [111] one growing linearly to a length L; the whisker B is also fast, having the same length, which during the growth was switched to another equivalent [111] direction. For the switching, an extra part is required, denoted by red in Figure 9. Therefore, extra dangling bonds are created for the same length, making this change unfavorable from the energetic point of view, especially at lower temperatures. The switching to the [111] direction is also shown in the schematic atomic representation viewed in the (110) section in Figure 9. The extra dangling bonds included in the red area are evident. It is worth noticing that the Ni-MILC at 413 °C is a very slow process permitting the atoms to find the lowest energy position minimizing the dangling bonds and resulting in straight whiskers as in VLS. This is not the case in the conventional Ni-MILC above 520 °C where significant SPC occurs. In this case, the <111> whiskers change their course frequently to another equivalent <111> direction, facilitating, in this way, the crystallization of the intermediate amorphous space by SPC growth as it is shown in Figure 2; see also M. Miyasaka et al. [14]. This is a consequence of the minimum action principle, namely the system takes the lower energy state in the minimum time.

### 3.6. Disturbed Ni-MILC at 413 °C

**Figure 10:**
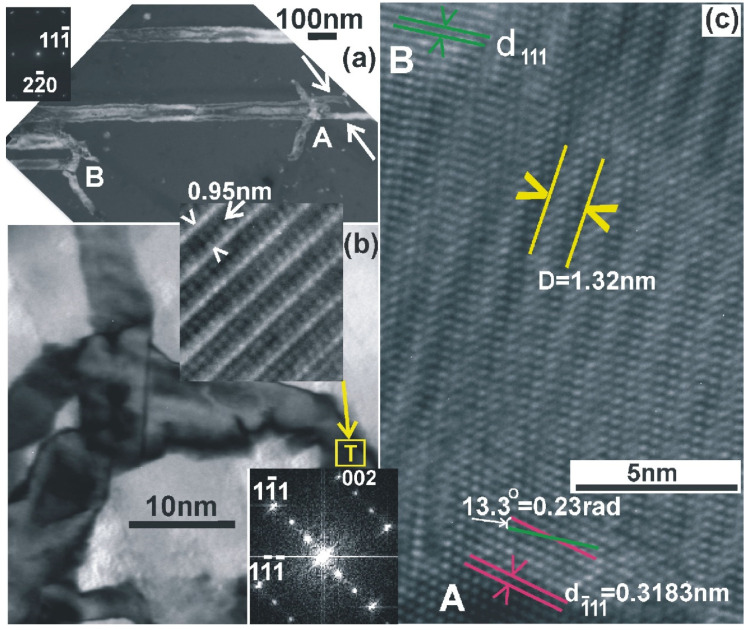
(**a**) DF-PVTEM micrograph showing disturbed nonlinear branches at the side-walls of the whiskers A and B. In the whisker A after the formation of the disturbed branches, the whisker was split into two parallel ones. In the case of whisker B, the growth was stopped. Both the whiskers were fast [111] types viewed in the (220) section. (**b**) The nonlinear branches consist of highly defected grains. In the area denoted by the letter T, Moiré pattern is observed with periodicity D_111_ = 3d_111_; these are formed by double diffraction of the electron beam in the matrix and an overlapping (111) twin; when viewed in the (110) section, this is confirmed by the Fast Fourier Transform shown in the inset. (**c**) Two overlapping grains, slightly misoriented A and B, are viewed in the (110) section, having the common (111) reflection in strong contrast. Moiré pattern of rotation type is observed, having periodicity of D_111_ = 1.38 nm, which corresponds to a misorientation of 1.34°.

In some cases, the Ni-MILC at 413 °C is disturbed so that short and thin nonlinear branches appear at the side-walls of the whisker as shown in the whiskers A and B in the DF-PVTEM micrograph in Figure 10a. It is speculated that the disturbance of the crystallization is due to contamination. Similarly, side-wall branches were observed due to contamination in nanowires grown by VLS [5]. In Figure 10a, in the area A, the formation of these branches is followed by splitting of the original whisker into two thinner ones, which are parallel to the original. In the region B, the crystallization stops after the formation of the side-wall branches. These branches are microcrystalline, consisting of grains having a mean size of 7 nm as shown in Figure 10b. In the grain denoted by the letter T in Figure 10b, a pattern with periodicity of three times the d_111_ spacing of the Si lattice, D = 3d_111_ = 0.95 nm, was observed along the direction [111]. This is confirmed by the extra spots in the Fast Fourier Transform (FFT) shown in the inset, in the bottom of Figure 10b. This is not a superlattice structure in Si; they are simply Moiré patterns which are formed by double diffraction of the electron beam in the Si matrix and an overlapping (111) type twin, i.e., a Σ3 type twin, viewed in the (110) section.

Very often, grains with different orientations overlap, giving Moiré pattern as shown in Figure 10c. The grain A viewed in the exact (110) section overlaps with the grain B which gives strong contrast from the (111) lattice planes which are rotated 13.3° (0.23 rad) in respect to the grain A, as shown in Figure 10c. Moiré pattern of the rotation type is formed in the overlapping area with periodicity D = d/θ = 0.3183 nm/0.23 = 1.38 nm. This was confirmed by measuring the periodicity of the Moiré patterns D in Figure 10c, which was found to be D = 1.32 nm. It is evident that these short and thin, highly defected whiskers were grown by the SPC process.

### 3.7. Impurities in the Si Whiskers

In the VLS method, many metals are used as catalysts for the growth of Si whiskers; from these, the most successful is gold. However, due to the contact between the liquid Au alloy and the whisker at a high temperature, the Si is inevitably contaminated by gold. This contamination increases the impurity level in the Si, degrading the electrical characteristics of the whisker [18]. Although there is a detailed paper on gold detection in Si nanowires [19], which says that incorporated gold does not influence in a significant way the carrier mobility in Si wires, when the surface density is low, the authors also mention that there are still challenging tasks in that technology. We also note that the gold concentration in the nanowires grown by the VLS method is significantly higher than the solubility limit of gold in the bulk Si, which is not very promising. Reference [19] points out that low surface density is essential; however, in Si, the lowest surface densities are found on (001) which is actually used in all CMOS technology because of the orientation of the wafers. In the case of the VLS grown nanowires, however, the side-walls are different and in some, saw-tooth faceting is observed in the (112) type side-walls of nanowires grown along the [111] direction by the VLS process [17]. Therefore, such Si whiskers are generally still not compatible with the current CMOS process [12]. Of course, the Ni-VLS process can be carried out as well and may give superior silicon wires with (111) orientation [20]; however, this requires a very high temperature of 1100 °C.

**Figure 11:**
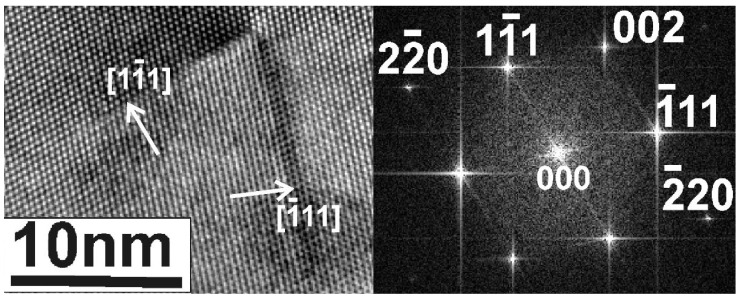
High-resolution TEM micrograph reveals a tetrahedral NiSi_2_ inclusion viewed in the (110) section as showing the related FFT in the lower left corner. The same defect is shown at low magnification in the upper right corner.

The whiskers grown by Ni-MILC are also contaminated by Ni metal as SIMS measurements in Si films grown at 575 °C reveal. Although the solubility limit of Ni in Si is very low (10^13^ atoms/cm^3^) at this temperature [21], a Ni concentration of 4 × 10^19^ atoms/cm^3^ or 0.08 at % Ni was measured. However, we have shown recently that the excess Ni in the Si whiskers is in the form of tetrahedral NiSi_2_ inclusions bounded by {111} coherent interfaces with the Si matrix [11]. The size of the inclusions ranges from a few atoms to 20 nm. The tetrahedral inclusions are formed by trapping NiSi_2_ clusters at the Si/NiSi_2_ interface during the whisker growth. The easy precipitation of NiSi_2_ in Si is attributed to the very low misfit, which is 0.4%. Due to the small misfit of the NiSi_2_ with the Si lattice and the small size of the tetrahedral inclusions, they cannot create misfit dislocations in their interfaces with the Si matrix. However, they do create some strain which gives a weak contrast in TEM. The high-resolution TEM micrograph in Figure 11 shows a V shape tetrahedral NiSi_2_ inclusion viewed in the (110) section. The NiSi_2_ inclusions are clearly visible in Z contrast; see Vouroutzis, N. et al. [11]. For the Si whisker grown by Ni-MILC at 413 °C, the average concentration of Ni is lower, 1.76 × 10^19^ Ni atoms/cm^3^ or in percentage 0.035 at %. The lower value of Ni concentration is attributed to the lowering of processing temperature. It is worth noticing that the amount of nickel inside the crystallized region depends on the annealing temperature, not on the annealing time [22]. It was shown that Si-whiskers grown by Ni-MILC are compatible with the CMOS processes and Thin Film Transistors were fabricated exhibiting very good performance [23]. The transistors presented in that publication were fabricated on polycrystalline Si after Ni-MILC above 540 °C where SPC is already also involved. Our nanowires were grown by Ni-MILC at 413 °C; where SPC is completely avoided is of the quality of the nanowires grown by VLS. Therefore, we believe that our material is useful from the technological point of view.

Nevertheless, the NiSi_2_ inclusions in the Si whiskers trap other metallic impurities there; this is a pathway for engineering impurities in Si [24].

In the Ni-MILC at 413 °C (i.e., the present experiments), the Ni concentration is 50% lower than at 575 °C, so we may expect additional improvement of the device behavior. This reduction of the Ni concentration is attributed to the lower process temperature. The exact influence of the NiSi_2_ inclusions on the electrical behavior of the nanowires is not known and further electrical characterization is required in order to reveal their influence on the device performance.

## 4. Conclusions

The VLS mechanism as well as Ni-MILC at 413 °C is a 1D crystal growth mechanism that is assisted by a metal catalyst. It results in the creation of whiskers and rods. The structural characteristics of the Si whiskers grown by Ni-MILC at temperature 413 °C were compared to those grown by the VLS method. The similarities are attributed to the suppression of the SPC at the side-bands of the whiskers in both methods. The whiskers are single crystalline, but those grown by the VLS require a single crystalline substrate and are grown perpendicular to it. In the case of Ni-MILC, the whiskers are grown parallel to an amorphous substrate; they emanate from the crystallographic facet of the NiSi_2_ grains. However, only whiskers which are parallel to the substrate can survive; from these, the prevalent ones are those which are grown fast due to the growth death competition mechanism [16]; these are the fast [111] whiskers.

The use of Au as the catalyst in VLS increases the impurity level in the bandgap of the Si whiskers, making them incompatible with the CMOS process. The Ni-MILC at 413 °C is a low-temperature process resulting in low Ni metal contamination level; in addition to the Ni forms, NiSi_2_ coherent inclusions in the Si whiskers act as traps for other metallic impurities, permitting the CMOS technology to be compatible with the Ni-MILC process. Moreover, the Si single crystalline whiskers grown by the low-temperature Ni-MILC technique can gain other applications in the nanomaterial subject. All the characteristics of the whiskers grown by VLS and Ni-MILC at 413 °C are summarized in Table 1.

## Figures and Tables

**Table 1:** Comparison of the Si whiskers grown by the VLS and the Ni-MILC at 413 °C methods.

Characteristics	VLS Method	Ni-MILC Method at 413 °C
Direction of growth	Perpendicular to the substrate	Parallel to the substrate
SPC growth	Suppressed	Suppressed
Temperature of the process	High	Low
Substrate	Single crystalline	Amorphous
Crystalline whiskers	This of substrate orientation	Fast [111]
Width of the whiskers	Polygonal with diameter of the hemispherical metallic catalysts	Depends on the size of the starting NiSi_2_ grain
Electrical characteristics	Not compatible with CMOS process due to metallic contamination	Compatible with CMOS process in spite of Ni contamination

## Data Availability

Authors provide experimental data for any reasonable request.

## References

[1] Puglisi R.A., Tanabe H., Chen C.M., Atwater H.A. (2000). Large-grained polycrystalline Si films obtained by selective nucleation and solid phase epitaxy. Mater. Sci. Eng. B.

[2] Qin M., Poon M.C., Fan L.J., Chan M., Yuen C.Y., Chan W.Y. (2002). Study of grain growth of polysilicon formed by nickel-induced-lateral-crystallyzation of amorphous silicon and subsequent high temperature annealing. Thin Solid Film..

[3] Mohiddon M.A., Krishna M.G., Dalba G., Rocca F. (2012). Transmission electron microscopy study of Ni–Si nanocomposite films. Mater. Sci. Eng. B.

[4] Anderson C., Kortshagen U. (2008). Seeding Solid Phase Crystallization of Amorphous Silicon Films with Embedded Nanocrystals. MRS Online Proc..

[5] Wagner R.S., Doherty C.J. (1966). Controlled Vapor-Liquid-Solid Growth of Silicon Crystals. J. Electrochem. Soc..

[6] Givargizov E.I. (1975). Fundamental aspects of VLS growth. J. Cryst. Growth.

[7] Radnóczi G.Z., Dodony E., Battistig G., Vouroutzis N., Kavouras P., Stoemenos J., Frangis N., Kovács A., Pécz B. (2016). Structural characterization of nanostructures grown by Ni metal induced lateral crystallization of amorphous-Si. J. Appl. Phys..

[8] Hayzelden C., Batstone J.L. (1993). Silicide formation and silicide-mediated crystallization of nickel-implanted amorphous silicon thin films. J. Appl. Phys..

[9] Su C.J., Huang Y.F., Lin H.C., Huang T.Y. (2012). Characterizations of polycrystalline silicon nanowire thin-film transistors enhanced by metal-induced lateral crystallization. Solid State Electron..

[10] Choi H.J., Gyu-Chul Y. (2012). Vapor–Liquid–Solid Growth of Semiconductor Nanowires. Semiconductor Nanostructures for Optoelectronic Devices.

[11] Radnóczi G.Z., Knez D., Hofer F., Frangis N., Vouroutzis N., Stoemenos J., Pécz B. (2017). Inclusions in Si whiskers grown by Ni metal induced lateral crystallization. J. Appl. Phys..

[12] Vouroutzis N., Stoemenos J., Frangis N., Radnóczi G.Z., Knez D., Hofer F., Pécz B. (2019). Structural characterization of poly-Si Films crystallized by Ni Metal Induced Lateral Crystallization. Sci. Rep..

[13] Hirsch P.B., Howie A., Nicholson R.B., Pashley D.W. (1965). Electron Microscopy of Thin Crystals.

[14] Miyasaka M., Makihira K., Asano T., Polychroniadis E., Stoemenos J. (2002). In situ observation of nickel metal-induced lateral crystallization of amorphous silicon thin films. Appl. Phys. Lett..

[15] Haji L., Joubert P., Stoemenos J., Economou N.A. (1994). Mode of growth and microstructure of polycrystalline silicon obtained by solid-phase crystallization of an amorphous silicon film. J. Appl. Phys..

[16] Kim M.S., Lee J.S., Kim Y.S., Joo S.K. (2006). The Effects of Crystal Filtering on Growth of Silicon Grains in Metal-Induced Lateral Crystallization. Electrochem. Solid State Lett..

[17] Ross F.M., Tersoff J., Reuter M.C. (2005). Sawtooth Faceting in Silicon Nanowires. Phys. Rev. Lett..

[18] Adhikari H., Marshall A.F., Goldthorpe I.A., Chidsey C.E.D., McIntyre P.C. (2007). Metastability of Au−Ge Liquid Nanocatalysts: Ge Vapor–Liquid–Solid Nanowire Growth Far below the Bulk Eutectic Temperature. ACS Nano..

[19] Allen J.E., Hemesath E.R., Perea D.E., Lensch-Falk J.L., Li Z.Y., Yin F., Gass M.H., Wang P., Bleloch A.L., Palmer R.E. (2008). High resolution detection of Au catalyst atoms in Si nanowires. Nat. Nanotechnol..

[20] Li F.J., Yuehua H., Shu W., Sam Z. (2020). Structure-sensitive principle in silicon nanowire growth. Thin Solid Film..

[21] Weber E.R. (1983). Transition metals in silicon. Appl. Phys. A.

[22] Cheng C.F., Poon V.M.C., Kok W., Chan M. (2003). Modeling of grain growth mechanism by nickel silicide reactive grain boundary effect in metal-induced-lateral-crystallization. IEEE Trans. Electron Devices.

[23] Lee S.W., Joo S.K. (1996). Low temperature poly-Si thin-film transistor fabrication by metal-induced lateral crystallization. IEEE Electron Device Lett..

[24] Fenning D.P., Newman B.K., Bertoni M.I., Hudelson S., Bernardis S., Marcus M.A., Fakra S.C., Buonassisi T. (2013). Local melting in silicon driven by retrograde solubility. Acta Mater..

